# The Science of Ageism: Two Steps Forward, One Step Back

**DOI:** 10.1093/geroni/igaa057.2222

**Published:** 2020-12-16

**Authors:** Jerin Lee, Natalie Shook

## Abstract

The past two decades have been marked by a rapidly aging population in the U.S. (U.S. Census Bureau, 2018), making prejudicial attitudes toward older adults (i.e., ageism) and the impact of such attitudes more relevant. As such, ageism researchers have worked tirelessly to not only understand this normalized and insidious form of bias, but also develop efforts to combat it. This symposium will feature four ageism researchers who will showcase both the growing pains and novel contributions of ageism research, ranging from the impact of ageism on psychological health to ageism interventions to issues related to the measurement of ageism. Specifically, Dr. Ayalon will present findings regarding difficulties with the assessment of exposure to ageism and the consequences of ageism for psychological well-being. Dr. Horhota will share research demonstrating challenges associated with confronting ageism. Dr. Levy will present a model showcasing factors associated with the reduction of ageism. Ms. Lee will discuss research findings examining the construct validity of several ageism measures. These talks highlight theoretical and real-world implications associated with the complex nature of ageism, providing important directions for enriching ageism research going forward.

